# The Qatari population’s genetic structure and gene flow as revealed by the Y chromosome

**DOI:** 10.1371/journal.pone.0290844

**Published:** 2023-09-01

**Authors:** Eida Khalaf Almohammed, Abdullah Hadi, Maha Al-Asmakh, Hayder Lazim

**Affiliations:** 1 Ministry of Interior of Qatar, Doha, Qatar; 2 Department of Biomedical Sciences, College of Health Sciences, QU Health, Qatar University, Doha, Qatar; 3 University of Central Lancashire Medical School, Preston, United Kingdom; 4 School of Medicine, Faculty of Health, Social Care and Medicine (FHSCM), Edge Hill University, Ormskirk, United Kingdom; Universita degli Studi di Roma Tor Vergata, ITALY

## Abstract

The Y-chromosome has been widely used in forensic genetic applications and human population genetic studies due to its uniparental origins. A large database on the Qatari population was created for comparison with other databases from the Arabian Peninsula, the Middle East, and Africa. We provide a study of 23 Y-STR loci included in PowerPlex Y23 (Promega, USA) that were genotyped to produce haplotypes in 379 unrelated males from Qatar, a country at the crossroads of migration patterns. Overall, the most polymorphic locus provided by the Promega kit was DYS458, with a genetic diversity value of 0.85 and a haplotype diversity of 0.998924. Athey’s Haplogroup Predictor tool was used to predict haplogroups from Y-STR haplotypes in the Qatari population. In a median-joining network, the haplogroup J1 predominance (49%) in Qatar generated a star-like expansion cluster. The graph of population Q-matrix was developed using Y-STR data from 38 Middle Eastern and 97 African populations (11,305 individuals), and it demonstrated a stronger sub-grouping of countries within each ethnic group and showed the effect of Arabs on the indigenous Berbers of North Africa. The estimated migration rate between the Qatari and other Arabian populations was inferred using Bayesian coalescence theory in the Migrate-n program. According to the Gene Flow study, the main migration route was from Yemen to Kuwait through Qatar. Our research, using the PowerPlex Y23 database, shows the importance of gene diversity, as well as regional and social structuring, in determining the utility of demographic and forensic databases.

## Introduction

**Fig 1:**
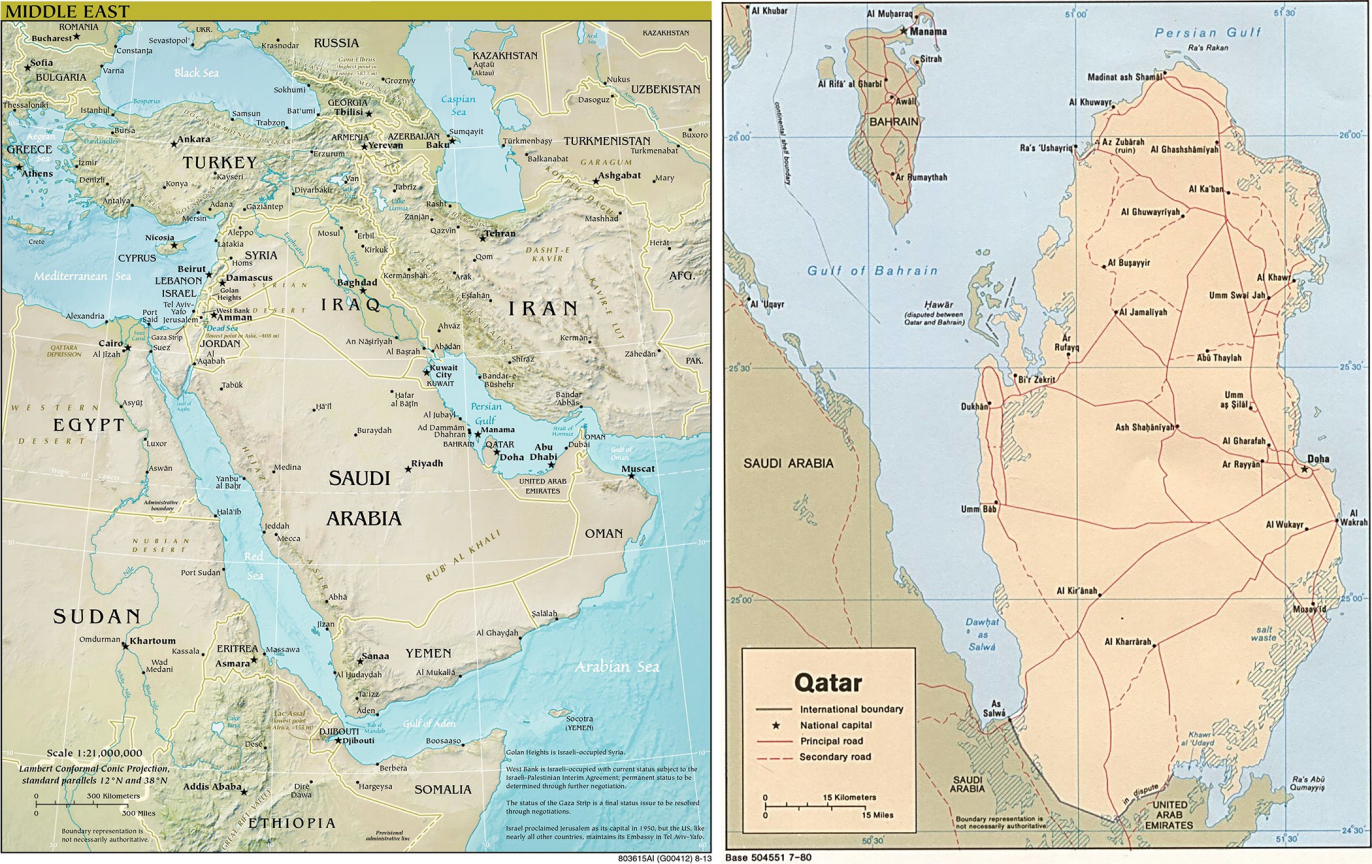
Map representing the geographic location of (A) The middle East (B) Qatar. Adopted from University of Texas library [4].

Qatar occupies the Qatar Peninsula, which extends northward from the Arabian Peninsula’s eastern edge into the Arabian Gulf and covers 11,427 km2 (Fig 1). Doha, the capital city, is located on the peninsula’s eastern edge. Qatar has a population of around 3,005,069 million people, including 300,000 nationals of various origins [1]. Qatar has a southern border with Saudi Arabia and a maritime border with Bahrain, the United Arab Emirates, and Iran. It is known, like other countries in the region, for its distinct population structure, which is characterized by a high consanguinity rate [2,3].

The Arabian Peninsula’s inhabitants can be divided into at least four genetic subpopulations that can show the region’s historical migration patterns: Bedouin, Iranians, South Asian, and African. This demographic pattern mirrored on the Qatari population, which included Iranians, Africans, and Bedouins. However, because of its extensive ancestry in the Arabian Peninsula, the Bedouin genetic subgroup was the most prominent [5–8].

The key position of Qatar on the Arabian Gulf was the main reason for the seasonal migration of Arab tribes from the Arabian Peninsula. It was a tricontinental nexus for human migration especially during the bidirectional dispersals between Eurasia and Africa [9].

Short tandem repeats on the human Y chromosome (Y-STRs) markers have useful characteristics for genetic applications such forensic evidence analysis, genealogy research, and historical investigations [10]. Additionally, the paternal inheritance pattern benefits Y-STRs in family research, ancestral origin investigations, and analysis of male/female DNA mixtures [11,12].

Y-STR markers on the Qatari population were studied previously by a limited number of markers. Qataris are organized into patrilineal descent groups, yet little has been done to study the population structure. A larger database of the Qatari population therefore needed to be established, for comparison with other Arabian Peninsula and Middle Eastern databases. The aim of this study is to investigate the genetic variability of the Arab population in Qatar utilizing 23 Y-STR loci from PowerPlex Y23 (Promega, USA) and to trace human migration in the Arabian Peninsula. Furthermore, we used STRUCTURE to examine the broad-scale population structure of the Middle East and Africa [13].

## Materials and methods

### Collection of Qatari population samples

A total of 379 saliva samples were collected anonymously from unrelated indigenous Qatari males Arabs from Doha. All samples used in this study were obtained for the purpose of the research and ethical approval was granted by the Ministry of Interior of Qatar and the University of Central Lancashire “Ref STEM454”. Informed consent forms were completed by all participants and authors had no access to information that could identify individual participants during or after data collection. The samples were collected taking into consideration relatedness, ensuring that all sample males were separated by at least three generations. The data were collected in June 2016 and made accessible for research purposes in August 2016.

### Inclusivity in global research

Additional information regarding the ethical, cultural, and scientific considerations specific to inclusivity in global research is included in the S1 Checklist.

### DNA extraction and quantification

The samples were extracted using the QIAamp^®^ extraction DNA Mini protocol (Qiagen Ltd, West Sussex, UK). The extraction procedure was followed in accordance with the manufacturer’s recommendations. The quantification of the collected samples was carried out using the Quantifiler^®^ Trio DNA Quantification kit (Thermo Fisher Scientific) according to the manufacturer’s instructions. All of the samples were quantified according to the manufacturer’s instructions on the 7500 Real-Time PCR System and analysed using the HID Real-Time PCR Analysis Software v1.2.

### PCR amplification and fragment analysis

The PowerPlex Y23 kit was used to generate 23 loci: DYS576, DYS389I, DYS448, DYS389II, DYS19, DYS391, DYS481, DYS549, DYS533, DYS438, DYS437, DYS570, DYS635, DYS390, DYS439, DYS392, DYS643, DYS393, DYS458, DYS385a/b, DYS456 and Y-GATA-H4. DNA amplification Reaction setup and thermal cycling were performed according to the procedures described in the PowerPlex Y23 kit User’s Manual. Thermal cycling was performed in Veriti^®^ 96-Well Thermal Cycler as recommended by the manufacturer (Thermo Fisher Scientific). PCRs were conducted using half of the recommended volume. Fragments were electrophoresed in eight capillaries (50-cm length) arrays on the ABI 3500 Genetic Analyser using the manufacturer’s recommended protocols (Thermo Fisher Scientific) filled with POP-4™ polymer. GeneMapper IDX software V1.4 was used for allele calling and interpretation.

### Statistical analyses

#### Forensic and population genetic parameters

The haplotype diversity for the Qatari population samples was evaluated by Nei’s formula [14], HD = n*(1 − Σ pi2)/ (n − 1) where n is the sample size and pi is the ith’s haplotype frequency. Haplotype frequency was calculated by the counting method. Genetic diversity (GD) was calculated as 1 − Σ pi2, where pi is the allele frequency. The match probability (MP) was calculated as Σ pi2, where pi is the frequency of the ith haplotype. The STRAF online tool was used to calculate haplotype diversity, GD, and MP [15]. Discrimination capacity (DC) was calculated by dividing the number of different haplotypes (h) by the total number of samples in a certain population (n) using the following formula: DC = h/n [16]. The haplotype match probability (HMP) was calculated as HMP = 1 –HD [17]. The Qatari population data were obtained using Y-STRs based on the PowerPlex Y23 kit, and compared to available published data for other close and distant populations.

The population genetic structure in our data was evaluated by the analysis of molecular variance (AMOVA). Molecular data were obtained for the Qatari population using Y-STRs based on the PowerPlex Y23 System and compared with the available data on other Middle Eastern populations [18–30]. Comparison with other datasets required reduction of the number of STRs to a shared set of 17, so that more Middle Eastern populations could be included in this analysis. Arlequin 3.5.2.2 software [31,32] was used to calculate the average pairwise differences between (PiXY) and within populations (PiX), in addition to the corrected average pairwise difference between populations (PiXY − (PiX + PiY)/2). More specifically, genetic distances between groups of males were quantified by Rst calculations based on Y-STR data and multi-dimensional scaling (MDS) plots. MDS analysis is also used to investigate genetic similarities between populations [33], and to visualize the variances of the genetic differences in Y-STR and between populations. The genetic matrices plots, the phylogenetic tree and the MDS plot were generated by using R statistical software version 4.0.

#### Qatari Y haplogroup assignment

In this research study, the methodology employed involved the use of full Y23 haplotypes to allocate haplotypes to their most likely haplogroup, a process which was facilitated through the utilization of Athey’s Haplogroup Predictor. It is pertinent to note that within the scope of this study, DYS543 and DYS533 were excluded from the data due to the unavailability of allele frequency data relating to these markers [34–36].

The batch program utilized 111 markers from the FTDNA set to conduct its prediction, employing the following set of criteria: a fitness score exceeding 15, a probability exceeding 85, and priors attuned to the Near East population. In several instances, the program failed to generate a prediction for certain samples as no haplogroup met the established criteria. Consequently, the haplotypes were subjected to manual examination, yielding results in some of the cases.

#### Median-joining networks Y-STR haplogroups analysis

The present study implemented Median-joining networks using specialized software, namely Network v5.0 and v2.1.2.5 [37]. To produce accurate and reliable results, intermediate alleles with repeat numbers were rounded off to the nearest integer. In line with the author’s recommendation, the constitutively duplicated loci (i.e., DYS385 a, b) were eliminated from the network construct. Notably, any missing data or deleted alleles were replaced with the code ’99’ in the input files, consistent with the standard practice of considering such data as missing.

#### Structure analysis

The structure of 38 Middle Eastern [18–30] and 97 African populations [28,38–56] was investigated using the programme STRUCTURE version 2.3.7 and an admixture model [13,57]. The graph of population Q-matrix was created using Y-STR data from 17 markers across 135 populations (11,305 individuals). The six new markers were removed from the PowerPlex Y23 System in order to include more Middle Eastern and African populations in this study.

The STRUCTURE HARVESTER program was employed to process the output and assess the probability values over a broad range of K values, alongside identifying the optimal number of genetic clusters that align with the data [58,59]. To consolidate the results, multiple iterative analyses of each dataset were aligned using CLUMPP [60,61], These aligned results were subsequently used to generate the graph of population Q-matrix using Distruct [60,62].

#### Migration rate in Qatari population

The Migrate software program employs a Bayesian inference framework to estimate several key population parameters, including effective population sizes, historical migration rates between varied populations under an asymmetrical migration model, and population divergences or admixture [63]. A data conversion tool, the PGD Spider tool, is utilized to format the data input file for analysis [64,65].

The Bayesian posterior probabilistic model is commonly utilized in the domain of population genetics to infer the parameters of a given model. Migrate, a widely-used software program, applies this approach to estimate population genetic parameters from the available genetic data. Migrate calculates the posterior probability of various population genetics parameters, such as migration rates and population sizes, by integrating possible relationships within the sample data. This is accomplished through the use of an expansion of the coalescent theory [66], which encompasses migration and/or population division [67]. Ultimately, this analytical approach provides a valuable tool for elucidating the complex genetic factors that contribute to population dynamics and evolution.

The gene flow was examined using four models in the Migrate software. Qatar was tested against five countries: Yemen, Saudi Arabia, Iraq, the United Arab Emirates and Kuwait. The present study used four distinct gene flow models to investigate migration patterns. The first model entailed unidirectional gene flow from one population to another. The second model accounted for divergence from a common ancestral population, while the third model incorporated both divergence from the ancestral population and ongoing immigration. The fourth model assumed that both populations belonged to the same panmictic population. To compare the relative strengths of the model fits, the log marginal likelihoods were utilized to calculate the Bayes factors. Magnitudes of the Bayes factors provided evidence for the degree of dissimilarity between the models.

To ensure thorough exploration of genealogic space, it is recommended that a single long chain is used in conjunction with a lengthy sampling increment of at least 1,000. This allows for efficient data exploration and facilitates the fitting of different genealogies; ultimately leading to more accurate confidence interval estimations.

In accordance with the prescribed methodology, a burn-in value of 5,000 discarded trees per chain was established. Subsequently, based on this value and the aforementioned parameter, each iteration within the sample set underwent an exhaustive evaluation process, totaling 5,000,000 assessments. The experimental approach employed the STATIC heating scheme, which entailed interchanging several chains, in an effort to achieve more comprehensive searches while maintaining an acceptable level of acceptance standards. To optimize the efficacy of the swapping methodology, four chains were utilized, corresponding to respective temperatures of 1.0, 1.5, 3.0, and 10,000, which were arranged in ascending order of thermal intensity. It should be noted that the initial temperature was maintained at a minimum of 1, in accordance with established guidelines [26].

## Results

The 23 Y-STRs profiled with PowerPlex Y23 kit were amplified for 379 Qatari males. S1 Table in S1 Data contains a full list of Qatari haplotypes, as well as other sample information; data are also accessible from YHRD, release v62 (Accession number YA004657). S2 Table in S1 Data contains the quantification information for Qatari DNA samples.

### Y-STR alleles and haplotype diversity within the Qatari population

For the purposes of calculating forensic parameters, 27 haplotypes with duplicated alleles were removed from the Qatari population, bringing the total number of haplotypes to 352. Allele frequency distributions of the 23-STR loci and the most frequent allele for each locus are presented in S3 Table in S1 Data for the 352 males of the population under study. Multiple alleles were observed for each locus ranging from 16 for DYS458 to five for DYS437. The difference between the overall repeat number at DYS389II and the repeat number at DYS389I was used to encode DYS389II alleles, which are marked as DYS389II.I in S1 Table in S1 Data.

**Fig 2:**
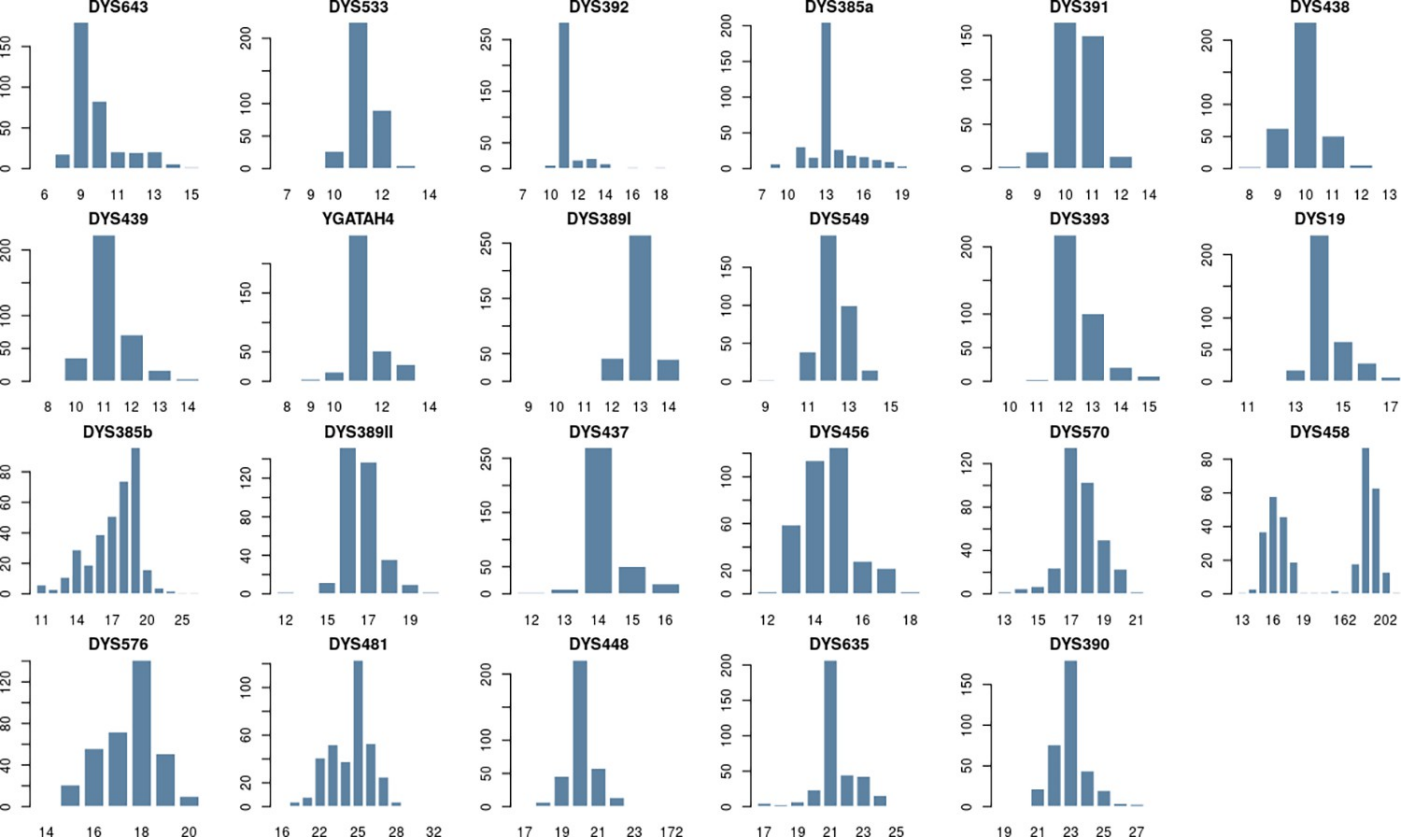
The alleles frequencies of the PowerPlex Y23 loci in the Qatari population. The difference between the overall repeat number at DYS389II and the repeat number at DYS389I was used to encode DYS389II alleles.

Genetic diversity and match probability values for each locus are presented in S4 Table in S1 Data. By far the most polymorphic locus was DYS458, with a genetic diversity value of 0.85; the least polymorphic locus was DYS392 with a genetic diversity value of 0.34. The diversity of four of the six newly added markers for the PowerPlex Y23 kit (DYS481, DYS576, DYS570 and DYS643) showed greater diversity than the Y filer loci, as can be inferred from the ranking of these loci (ranks 3, 4, 5 and 7); the other two loci (DYS549 and DYS533) did not show such a high diversity and their ranks were 12 and 19 respectively. The alleles frequencies of the Qatari population shown in Fig 2. Gene diversity (GD) values exceeded (0.5) for all 19 markers, and (0.6) for 11 markers (S4 Table in S1 Data). The online tool STRAF [68] was used to calculate the forensic parameters.

A total of 327 were the unique haplotype and the DC for the kit was 86.2% for the Qatari population. The haplotype diversity for the Qatari population was 0.998924.

We compared the performance of the PowerPlex Y23 kit to the previous Y-filer kit (AmpFLSTR™ Yfiler™ PCR Amplification Kit (Applied Biosystems™)) by eliminating the six newly added markers, and the number of distinct haplotypes was reduced to 289 and the DC was reduced to 76.2%. Furthermore, haplotype diversity dropped to 0.995498.

### Athey haplogroup prediction results for Qatari samples

Y-STR haplotypes for 379 male samples were obtained for Y-STRs. STR haplogroups were determined using the haplogroup prediction tool by Athey. The majority (48.5%) of 379 Y-haplogroups was assigned to haplogroup J1, a branch of which was the predominant Y-chromosome haplogroup of populations in the Arabian Peninsula; this was followed by J2 (12.9%), R1a (7.7%), E1b1b (5.3%), E1b1a (5%), T (4%), L (4%), G2a (2.1%), R1b (1.1%), and less than 1% for the rest of the haplogroups (S5 Table in S1 Data). S1 Table in S1 Data contains a full list of haplogroups predicted from STR haplotypes; using the prediction Athey tool. Some 16 unpredicted haplotypes (UP) were not assigned to any of the haplogroups.

### Median joining network for Qatari population

**Fig 3:**
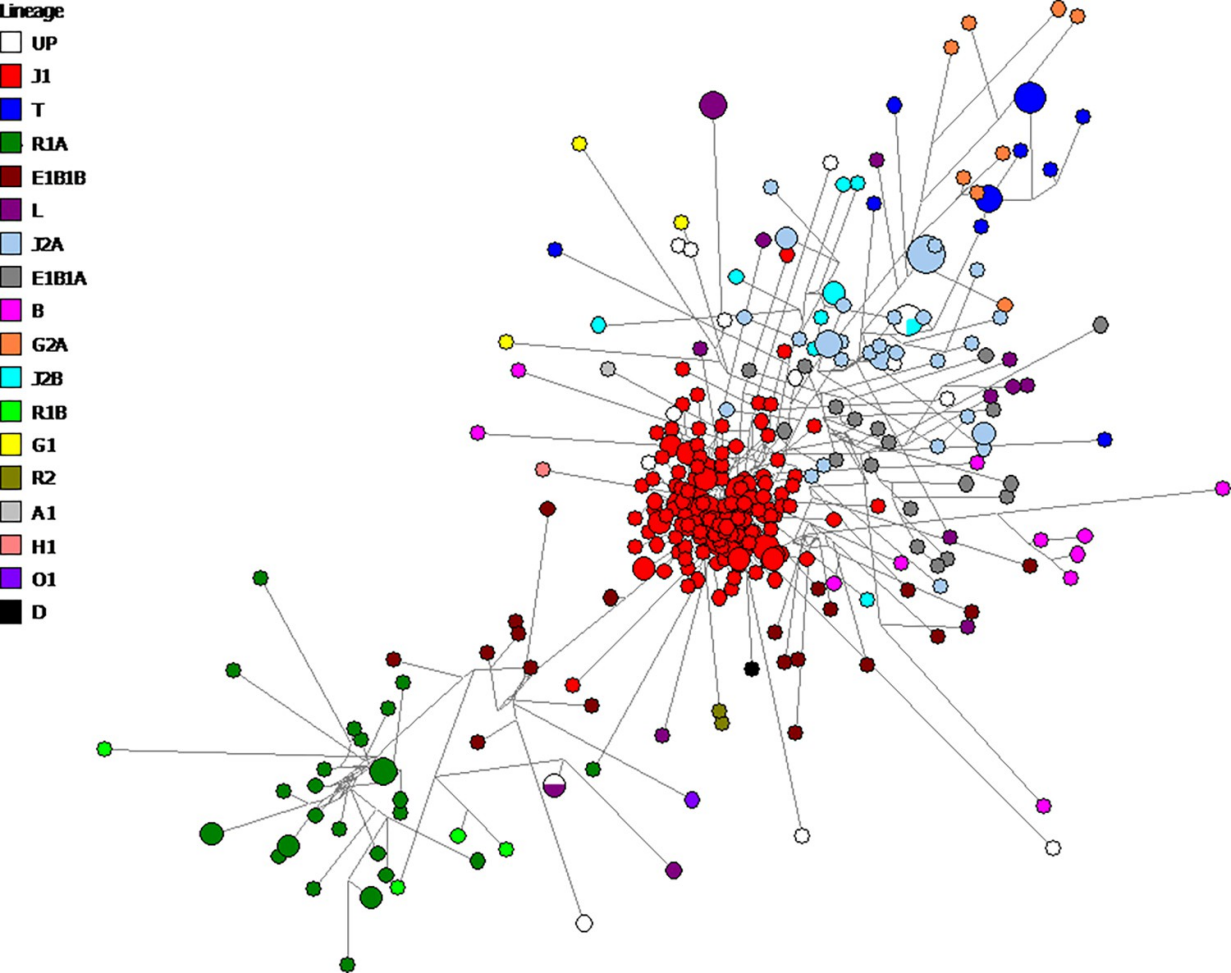
Median-joining network of Y-STR haplotypes for Qatari population distribution of predicted haplogroups. Circles represent haplogroups, with an area proportional to sample size, and lines between them proportional to the number of mutational steps. Haplogroup categories represented in different colours are explained in the top left legend. The network’s major cluster haplogroups are assigned to J1 (48.5% of the total Qatari population). UP: unpredicted haplogroups.

In order to understand the relationships between Y-STR haplotypes in the dataset, this study constructed a median-joining network (Fig 3). Based on Athey predictions, haplogroups were assigned within the network major as predicted to form clusters. However, the dominant feature is a central star-like cluster of closely linked haplogroups; the red circles represent J1 haplogroups (48.5% of the total sample, S5 Table in S1 Data), suggesting a recent expansion of this haplogroup. It was noticed that 98.9% (182 haplotype) of the Y-chromosomes carrying the DYS458 microvariants were located within haplogroup J1 (S1 Table in S1 Data).

### Populations’ structure and admixture

**Fig 4:**
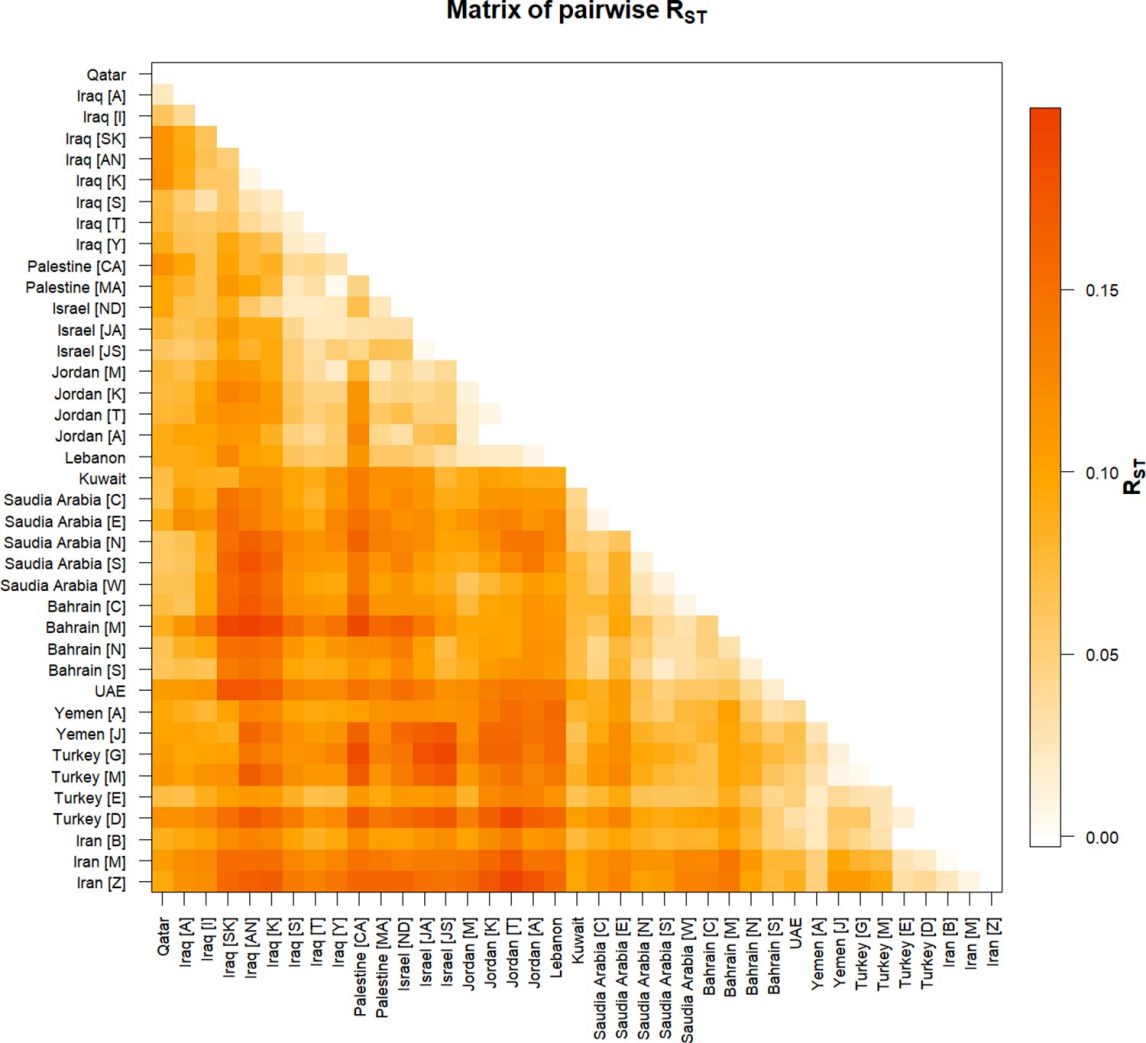
The matrix of pairwise genetic distance Rst of Y-STR between the Qatari population and the other Middle Eastern populations based on 17 Y-STR markers. The Qatari population was compared to 38 regions and ethnic groups in the Middle east. This matrix was generated using Arlequin 3.5.2.2 software. Bahrain [C: Capital]; Bahrain [M: Muharraq]; Bahrain [N: Northern]; Bahrain [S: Southern]; Iran [B: Birjand, South Khorasan]; Iran [M: Mashhad, Razavi Khorasan]; Iran [Z: Zahedan, Sistan and Baluchestan]; Iraq [A: Arab]; Iraq [AN: Arab North]; Iraq [I: Iraqi]; Iraq [K: Kurd]; Iraq [S: Syriac]; Iraq [SK: Sorani Kurd]; Iraq [T: Turkmen]; Iraq [Y: Yazidi]; Israel [JA: Jewish, Ashkenazi]; Israel [JS: Jewish, Samaritans]; Israel [ND: Northern, Druze]; Jordan [A: South, Aqaba]; Jordan [K: South, Karak]; Jordan [M: South, Ma’an]; Jordan [T: South, Tafila]; Palestine [CA: Christian Arab]; Palestine [MA: Muslim Arab]; Saudi Arabia [C: Central]; Saudi Arabia[E: east]; Saudi Arabia [N: north]; Saudi Arabia [S: south]; Saudi Arabia [W: west]; Turkey [D: Dogukoy]; Turkey [E: Eskikoy]; Turkey [G: Gocmenkoy]; Turkey [M: Merkez]; UAE: United Arab Emirate; Yemen [A: Arab]; Yemen [J: Jews].

A pairwise matrix plot of R_ST_ Distances generated to compare the Qatari population with 38 other populations using 17 loci (Fig 4 and S6 Table in S1 Data). The closest relationship is found between Qatar and Iraq [Arab] (R_ST_ = 0.02542), then Saudi Arabia [south] (R_ST_ = 0.05873), and Saudi Arabia [north] (R_ST_ = 0.06009). The farthest relationship is found between Qatar and Iraq [Kurd], Turkey [Dogukoy] and Palestine [Christian Arab] with R_ST_ values (0.1177, 0.11777 and 0.11983) respectively.

The highest R_ST_ pairwise value was between Bahrain [Muharraq] and Iraq [Arab North] (R_ST_ = 0.19963), whereas the lowest pairwise R_ST_ value was (R_ST_ = -0.00295) between Jordan [South, Aqaba] and Jordan [South, Karak].

**Fig 5:**
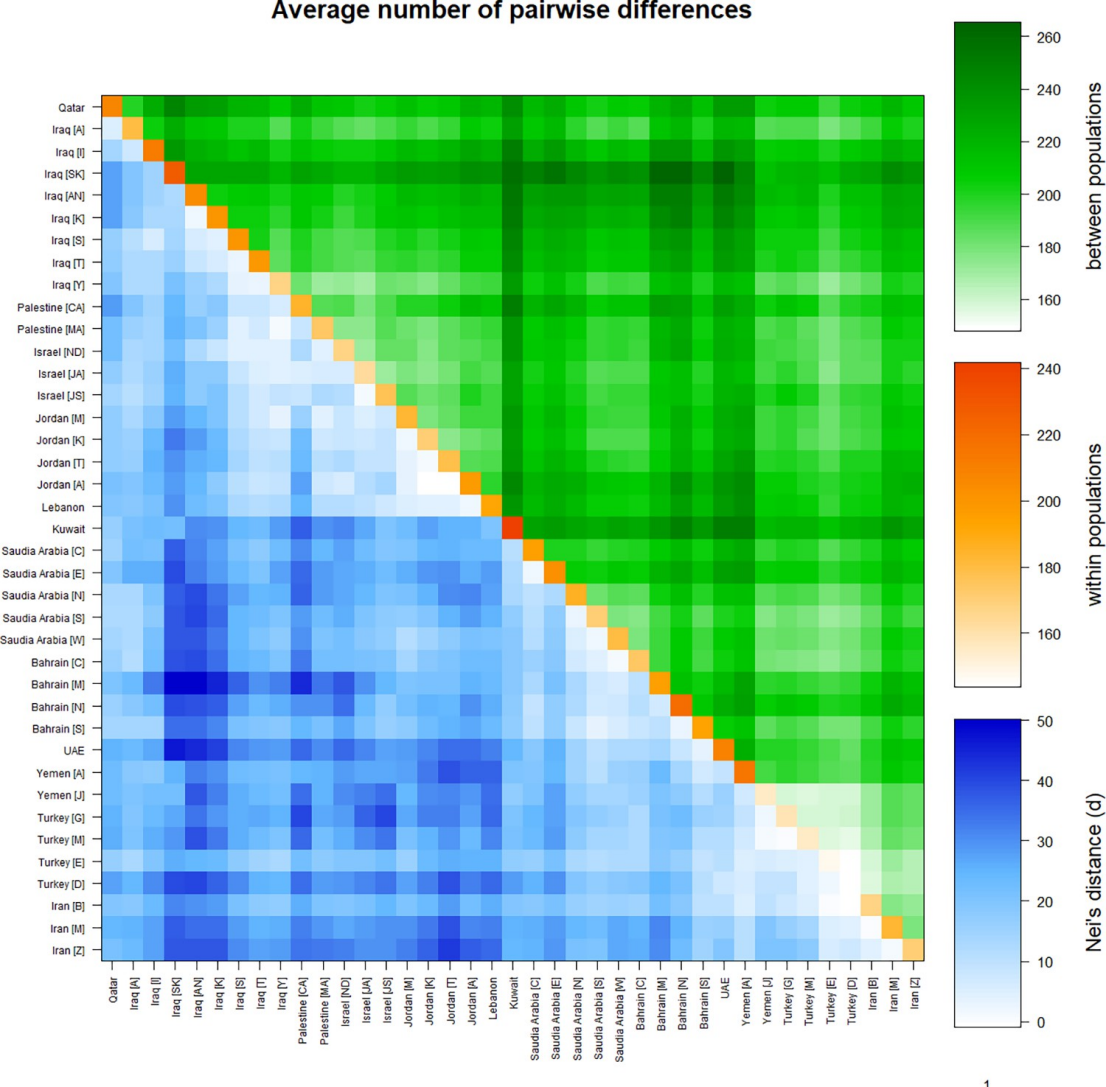
Matrix plot showing population average pairwise differences based on 17 loci. The area above the diagonal (green) shows the average number of pairwise differences between populations (PiXY); the diagonal (orange) shows the average number of pairwise differences within population (PiX); and below the diagonal (blue) shows the corrected average pairwise difference (PiXY-(PiX+PiY)/2). The scale of differences is shown on the right side of the matrix. This matrix was generated using Arlequin 3.5.2.2 software. Bahrain [C: Capital]; Bahrain [M: Muharraq]; Bahrain [N: Northern]; Bahrain [S: Southern]; Iran [B: Birjand, South Khorasan]; Iran [M: Mashhad, Razavi Khorasan]; Iran [Z: Zahedan, Sistan and Baluchestan]; Iraq [A: Arab]; Iraq [AN: Arab North]; Iraq [I: Iraqi]; Iraq [K: Kurd]; Iraq [S: Syriac]; Iraq [SK: Sorani Kurd]; Iraq [T: Turkmen]; Iraq [Y: Yazidi]; Israel [JA: Jewish, Ashkenazi]; Israel [JS: Jewish, Samaritans]; Israel [ND: Northern, Druze]; Jordan [A: South, Aqaba]; Jordan [K: South, Karak]; Jordan [M: South, Ma’an]; Jordan [T: South, Tafila]; Palestine [CA: Christian Arab]; Palestine [MA: Muslim Arab]; Saudi Arabia [C: Central]; Saudi Arabia[E: east]; Saudi Arabia [N: north]; Saudi Arabia [S: south]; Saudi Arabia [W: west]; Turkey [D: Dogukoy]; Turkey [E: Eskikoy]; Turkey [G: Gocmenkoy]; Turkey [M: Merkez]; UAE: United Arab Emirate; Yemen [A: Arab]; Yemen [J: Jews].

The average pairwise differences among Middle Eastern populations were calculated using 17 loci. This average pairwise difference was calculated to show the genetic differences between the 38 Middle Eastern populations and within these populations, in addition to among populations using Nei’s distance (Fig 5 and S7 Table in S1 Data).

The highest corrected average pairwise value using Nei’s distance between populations was (50.20693) between Bahrain [Muharraq] and Iraq [Sorani Kurd]. Jordan [South,Karak] and Jordan [South, Aqaba] had the lowest value (-0.9117).

Kuwait had the highest average pairwise difference value within populations (241.8143), while Turkey [Dogukoy] had the lowest value (143.64482).

The highest average pairwise value between populations was (265.31249) between UAE and Iraq [Sorani Kurd], while the lowest value was (147.99659) between Turkey [Eskikoy] and Turkey [Dogukoy].

**Fig 6:**
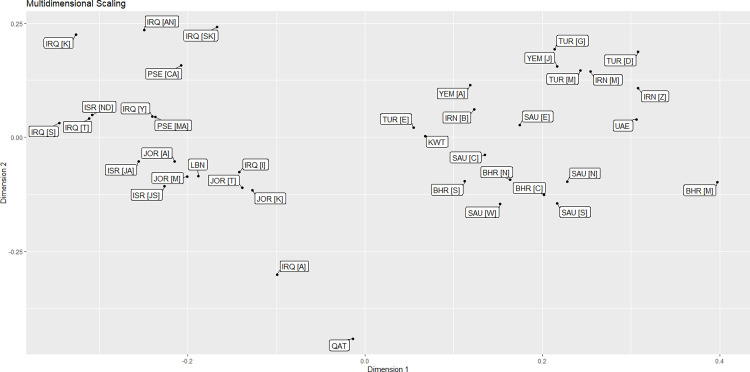
Multidimensional scaling (MDS) plots comparing the results of Qatari and Arabian Peninsula populations based on 17 Y-STR markers. The Qatari population was compared to 38 regions and ethnic groups in the Middle East. The MDS figure was generated using R statistical software version 4.0.3. BHR [C]: Bahrain [Capital], BHR [M]: Bahrain [Muharraq], BHR [N]: Bahrain [Northern], BHR [S]: Bahrain [Southern],IRN [B]: Iran [Birjand, South khorasan], IRN [M]: Iran[Mashhad, Razavi khorasan], IRN [Z]: Iran [Zahedan, Sistan and Baluchestan], IRQ [A]: Iraq [Arab], IRQ [AN]: Iraq [Arab North], IRQ [I]: Iraq [Iraqi], [K]: Iraq [Kurd], IRQ IRQ [S]: Iraq [Syriac], IRQ [SK]: Iraq [Sorani Kurd], IRQ [T]: Iraq [Turkmen], IRQ [Y]: Iraq [Yazidi], ISR [JA]: Israel [Jewish, Ashkenazi], ISR [JS]: Israel [Jewish, Samaritans], ISR [ND]: Israel [Northern, Druze], JOR [A]: Jordan [South, Aqaba], JOR [K]: Jordan [South, Karak], JOR [M]: Jordan [South, Ma’an], JOR [T]: Jordan [South, Tafila], KWT: Kuwait [Arab], LBN: Lebanon [Lebanese], PSE [CA]: Palestine [Christian Arab], PSE [MA]: Palestine [Muslim Arab], SAU [C]: Saudi Arabia [Central], SAU [E]: Saudi Arabia [east], SAU [N]: Saudi Arabia [north], SAU [S]: Saudi Arabia [south], SAU [W]: Saudi Arabia [west], TUR [D]: Turkey [Dogukoy], TUR [E]: Turkey [Eskikoy], TUR [G]: Turkey [Gocmenkoy], TUR [M]: Turkey [Merkez], UAE: UAE [Arab], YEM [A]: Yemen [Arab], YEM [J]: Yemen [Jews].

In the MDS and genetic distances between populations for Y-filer (17 markers) were calculated between all populations using the R statistical program version 4.0.3, to display the relationships among the 38 Middle Eastern populations (Fig 6). Two Middle Eastern clusters have been identified in the plot’s first dimension. The first cluster includes the upper Arabian Peninsula, Palestine, Israel, Iraq, and Jordan, as well as Qatar. The second cluster included all of the remaining Middle Eastern populations.

The second dimension of the plot shows four clusters located in different quarters of the MDS were created. Iraq (Iraqi), Lebanon Israel (Jewish, Ashkenazi, Samaritans), Jordan (Ma’an, Karak, Tafila, Aqaba), fell into one cluster in the lower left quadrant, while Iraq (Arab) and Qatar were further away to the lower part of this quadrant. The rest of the Iraqi ethnic groups (Arab north, Sorani Kurd, Kurd, Yazidi, Syriac and Turkman) were clustered together with Palestine (Christian and Muslims) in the upper left quadrant. Saudi Arabia (north, south, west, central) formed a cluster with Bahrain (north, central, south), Iran (Birjand, South Khorasan), Yemen (Arab) and Turkey (Eskikoy) extending from the lower right to the upper right quadrant. The fourth cluster occupied the upper right quadrant and formed by Turkey (Gocmenkoy, Merkez, Dogukoy), Iran (Zahedan, Sistan and Baluchestan), Yemen (Jews) and Saudi Arabia (east).

**Fig 7:**
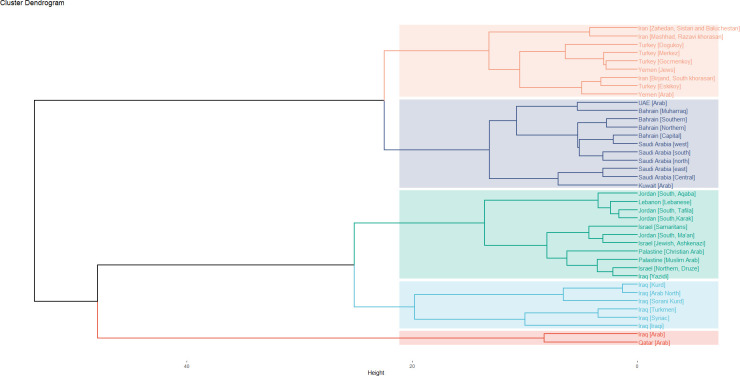
Dendrogram of the 38 populations. Five clusters (K = 5) were created. Qatar with other Middle Eastern populations fell into one cluster. This dendrogram was generated using R statistical software version 4.0.3.

Phylogenetic tree was generated using R statistical program version 4.0.3. The 38 Middle Eastern populations were clustered using the R statistical programme version 4.0.3. (4,471 haplotypes). Six clusters (K = 6) were identified. As shown in Fig 7, Qatar clustered with the Iraqi [Arab] population. Except for the Iraq [Yazidi], all Iraqi ethnic groups form a second cluster. Iraq [Yazidi] was clustered with the Levantine countries of Jordan, Palestine, Lebanon, and Israel.

The third cluster consists of four gulf countries: the UAE, Saudi Arabia, Kuwait, and Bahrain, while the rest of the Middle Eastern countries comprise the sixth cluster.

**Fig 8:**
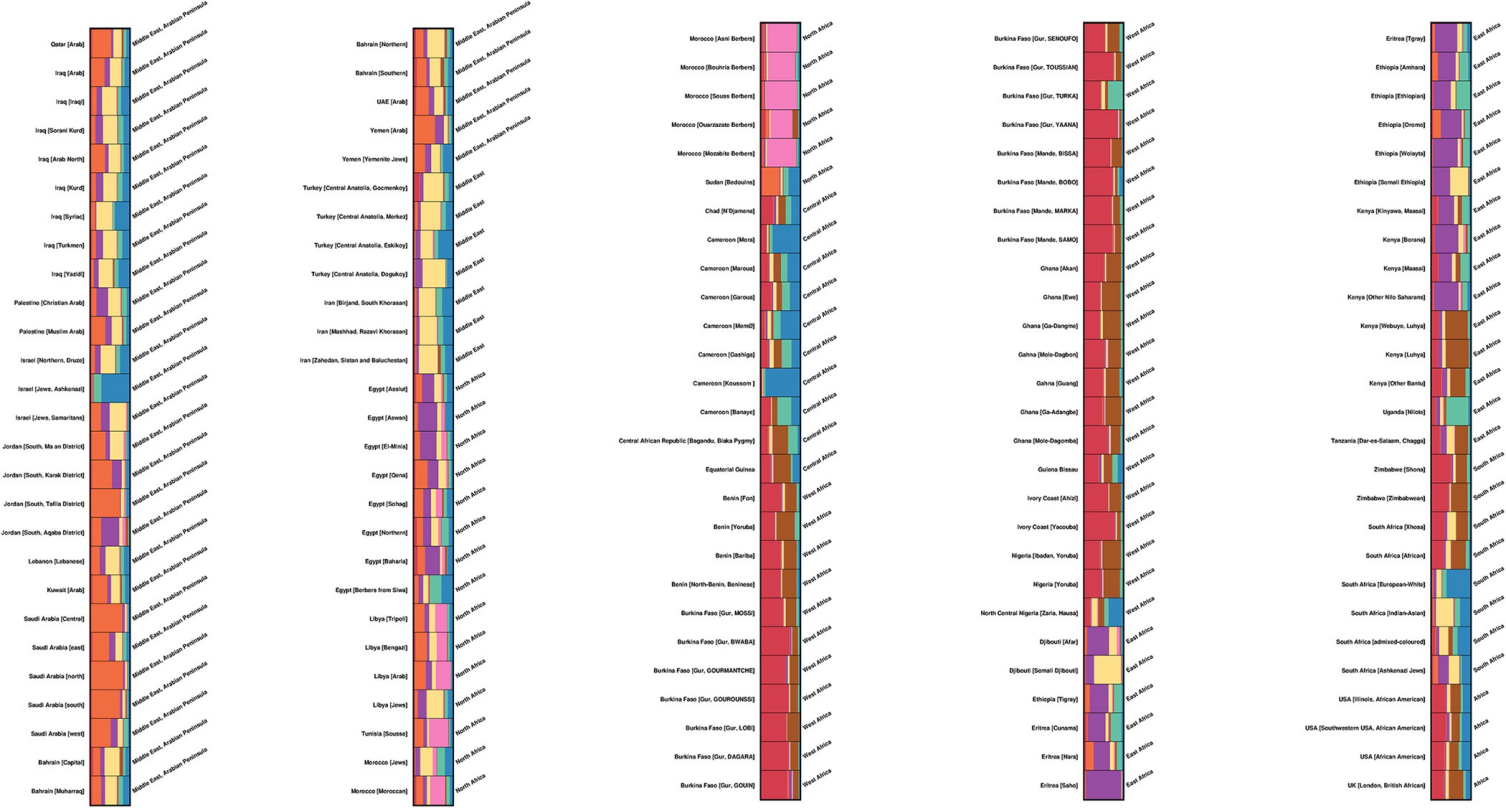
The graph of population Q- matrix of the Y-STR haplotypes using 17 STR markers from 135 Middle Eastern and African populations (11,305 individuals) showing 8 clusters (K = 8).

The Y-STR graph of population Q-matrix (Fig 8) revealed eight clusters using 17 STR markers from 135 African and Middle Eastern populations (11,305 individuals). The identified clusters of individuals in the Y-STR corresponded to specific geographical regions without overlap and revealed a stronger sub-grouping of countries within each population.

The populations of the Middle East form their own cluster. One notable difference was the Jewish Ashkenazi population. The north Africa cluster demonstrates that Egyptians are distinct from other north African populations. Except for the Libyan and Moroccan Jews, who showed similar clusters to the Middle Eastern populations, Libya, Tunisia, and Morocco shared the same pattern. Sudan’s population structure is similar to that of central Africa.

With the exception of Cameroon, Central African populations form a consistent cluster. The pattern then gradually shifted towards the west African populations, with the exception of Tanzania and two Kenyan populations (Luhya and other Bantu), which showed the similar clusters as the west African populations. The South African population has its own cluster, with the exception of people of different racial and ethnic backgrounds, including European, Asian, and Ashkenazi Jews. The four African population samples collected outside of Africa, in the United States and the United Kingdom, were not assigned to any of the African regions and are included at the end of the population Q-matrix graph. Their cluster pattern, however, appeared to be very similar to that of west and south African populations.

The populations of Jews were seen in three distinct parts of the admixture plot. We have Ashkenazi, Samaritan, and Yemenite Jews in the Middle East; north African Jews in Libya and Morocco; and Ashkenazi Jews in south Africa. With the exception of Ashkenazi Jews in the Middle East, all Jewish populations share the same structure as the Middle Eastern populations.

Despite sharing a pink colouring with the North African Arabs of Libya, Tunisia, and Morocco, the Berbers of Morocco (Asni, Bouhria, Souss, Ouarzazate, and Mzabite) may be distinguished from them by their different genetic profile. The Egyptian Berbers of Swia, on the other hand, shared certain characteristics with the Middle Eastern cluster.

### Estimation of migration rate in the Qatari population

**Fig 9:**
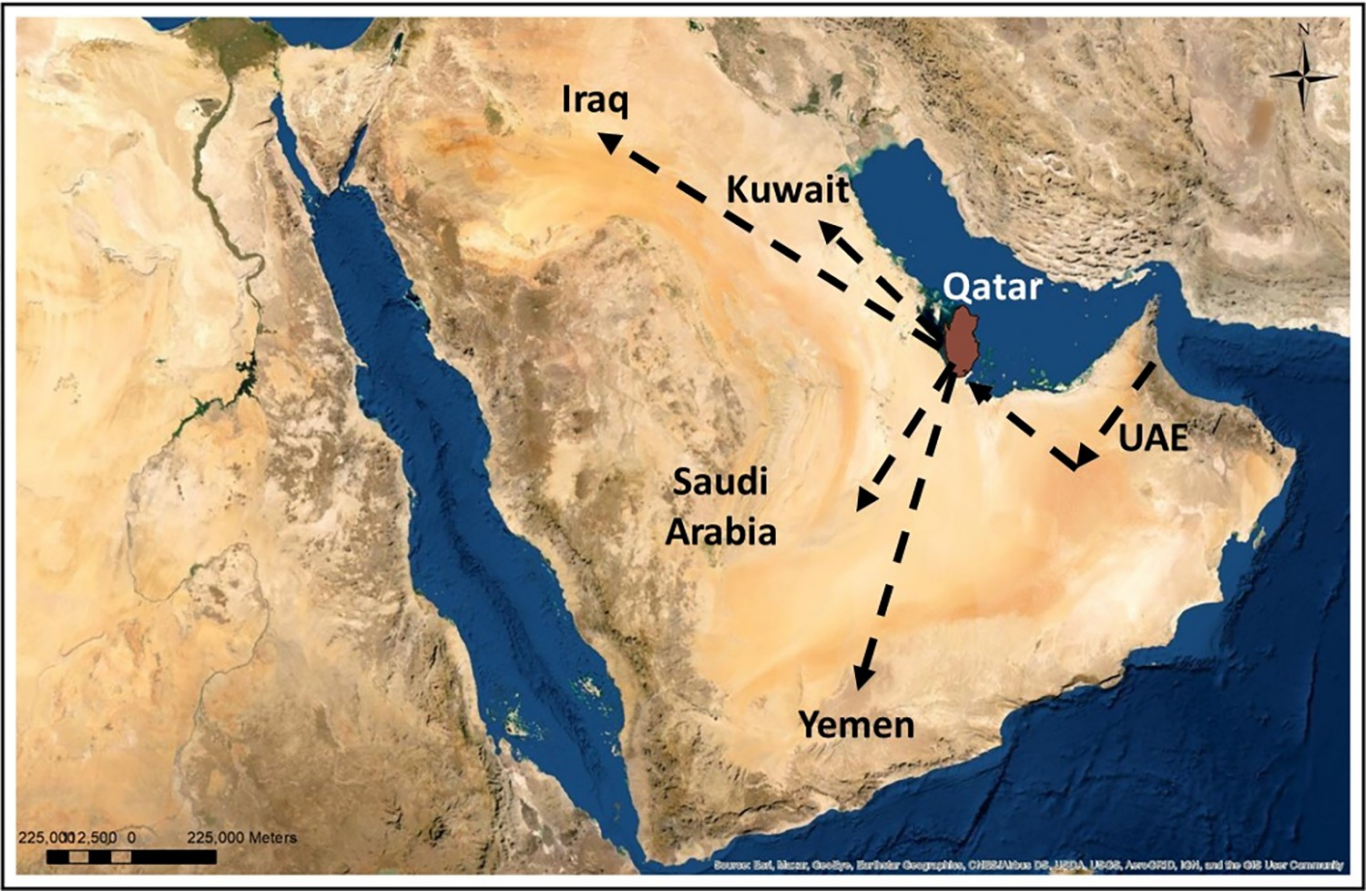
The most dominant migration routes between Qatar and five other countries. UAE → Qatar, Qatar→ Saudi, Qatar → Yemen, Qatar → Kuwait, Qatar→ Iraq. Map created in ArcGIS software [69].

Four migration model routes were used to investigate Qatari population movements and their impact on the Arabian Peninsula. Between Qatar and the five countries of the UAE, Iraq, Kuwait, Saudi Arabia, and Yemen, five routes were examined. For each route, four models were used, and the results revealed that model 2 was the most significant, as shown in S8 Table in S1 Data. To determine which country has the greatest impact on Qatar, the most dominant model for each route was used (Fig 9 and S8 Table in S1 Data).

Qatari inhabitants exhibit two bidirectional migration trends, the first from Qatar to Yemen (Log (ml) = -2244.24), Saudi Arabia (Log(ml) = -2465.67), Kuwait (Log (ml) = -2384.68), and Iraq (Log (ml) = -2496.75), all of which show a minor predominance. The second migratory route was from the UAE to Qatar (Log (ml) = -2444.35), which was slightly more prevalent. The most likely migration routes among these five were between Qatar and both Yemen and Kuwait. However, the route Yemen, Qatar, and Kuwait is considered part of the Arabian Peninsula’s coastal migration path (Fig 9).

## Discussion

In the current investigation, an analysis was conducted on Y-chromosomal STR haplotypes utilizing the PowerPlex®Y23 kit (Promega Corporation). As anticipated, this kit demonstrated a robust discriminative power suitable for forensic applications. The robustness of the locus outcomes generated can be attributed to the greater number of markers employed in the kit and the intentional selection of markers with a heightened capacity for differentiation. In particular, it is noteworthy that three out of the top five Y-STRs exhibiting greatest diversity in this examination (DYS481, DYS570, and DYS576) were unique to the PowerPlex®Y23, and in agreement with global research findings conducted across various meta-populations [28,70].

With a proportion of 51.1%, the Qatari population demonstrates the third highest identified frequency of microvariant alleles at the DYS458 marker, trailing behind Saudi Arabia (70%) [25], and Yemen (65%) [YHRD accession YA005529]. This high prevalence of microvariants is particularly noteworthy, given that it is indicative of the Middle Eastern populations, a group with which the Qatari population shares a common ancestry and geographic proximity. The presence of microvariant alleles offers the potential to boost the discriminatory capacity and probative value of DNA profiling, thereby facilitating the identification of populations’ haplogroups.

The present investigation determined that haplotypes containing microvariants located at DYS458 among the Qatari population were notably correlated with haplogroup J1. This observation agrees with recent research conducted on Y-chromosome studies within the Arabian Peninsula [18,20,25,26].

The dominancy of the haplogroup J1 in the Arabian Peninsula can be elucidated through previous investigations which have established a correlation between haplogroup J1 and aridity in the Arabian Peninsula, as well as its association with the speakers of Semitic tongues, notably Arabic. In addition to its prevalence among the Middle Eastern populations, this haplogroup has also been traced among Central Asian and South Asian communities. Furthermore, its detection has been documented in contemporary West Asia, North Africa, the Horn of Africa, and Southern Europe [71–73]. Numerous studies have extensively documented the prevalence of haplogroup J1 in the Arabian Peninsula, including Yemeni (72.6%), Saudi Arabian (71%), UAE (34.8%), Omani (38%), and Iraqi (36.6%) populations. However, it is noteworthy that the presence of the J1 haplogroup appears to be relatively scarce within various Middle Eastern populations, such as Bahraini (23%), Lebanese (12.5%), and Turkish (8.99%) populations [18,25,26,74].

The founder effect presents an additional factor that contributes to the prevalence of the J1 haplogroup, which is accompanied by the process of genetic drift. Moreover, the region in question exhibits significant levels of non-random mating, with cultural practices that foster patterns of patrilocality and polygamy, thereby promoting the preservation of male lineages within the area. Additionally, consanguineous marriages—including those between first cousins—are a notable feature of Middle Eastern societies due to long-standing Muslim traditions, leading to inbreeding phenomena that serve to propagate a particular patrilineage [74].

In scholarly discourse, researchers have recently endeavoured to compare Middle Eastern populations by utilizing MDS plots and R_ST_ measures. It is noteworthy, however, that these prior works have only limitedly explored a reduced range of 9–11 Middle Eastern populations for comparative analysis, as evidenced by studies such as those outlined in references [18,24,30]. To the best of our knowledge, the present study represents the first empirical investigation to undertake a comprehensive examination of a significantly larger sample of 38 distinct Middle Eastern ethnic and regional populations.

The findings of the Multidimensional Scaling (MDS) analysis indicated that the nations located within the Arabian Peninsula exhibited a distinct clustering pattern. However, the present investigation has revealed that the Qatari demographic shares greater proximity with the Iraqi Arab population and has formed a separate cluster when distinct from the remaining populations residing within the Arabian Peninsula. Notably, the uncovering of valuable archaeological evidence, such as diagnostic pottery sherds belonging to the Ubaid period (6000 B.C. to 4000 B.C.), has further amplified the existence of an interconnected relationship between the regions of the Gulf and Mesopotamia [75,76].

In recent years, much attention has been paid to populations structure analyses of the Y chromosome. As such, with regards to the Middle East, it is worth noting that there have been two studies conducted on the said topic. The first of these studies, conducted by Lazim et al., undertook a global analysis of Y-STR in 134 populations worldwide, encompassing 21,323 individuals. While this work was able to identify nine specific clusters using 19 markers, its limitations included a lack of representation, with only seven Middle Eastern and ten African populations examined [26].

In contrast, the second study exclusively examined 23 Middle Eastern populations totalling 3833 individuals and provided insight into four distinct clusters, using 17 markers [20]. However, despite the merits of these previous studies, it has been suggested that they were ultimately limited in scope, failing to provide an inclusive or comprehensive understanding of the genetic makeup of Africa and the Middle East.

It is in this context that our study presents a significant contribution to the forensic field. In seeking to build upon the insights provided by previous researchers, our study adopted the population Q-matrices of the Y-STRs and harnessed its full potential in a carefully crafted examination of 97 African and 38 Middle Eastern populations. Our study drew upon 17 STR markers in our identification of eight clusters, culminating in an extensive analysis of 11,305 individuals. Ultimately, our work represents a more thorough and precise exploration of the genetics of Africa and the Middle East as compared to earlier studies.

Despite the inclusion of two distinct geographic regions, Africa and the Middle East, and the implementation of 17 markers, the population Q-matrix yielded a remarkable graph total of 8 clusters (K = 8). This outcome suggests an unexpectedly high number of distinct geographic and population relationships. It is worth noting that in the preceding worldwide investigation, 9 clusters were discovered through the use of 19 markers. [26]. This could be attributed to the broader scope of the survey’s demographic size and ethnic composition.

The African continent boasts of striking genetic, linguistic, cultural, and phenotypic diversity. With more than 2000 distinct ethno-linguistic groups, African languages make up almost one-third of the world’s spoken languages. The African populations exhibits a wide range of subsistence patterns, including various agriculture practices, pastoralism, and hunting and gathering. Notably, African languages have been classified into four primary language families, namely Niger-Kordofanian, Afro-Asiatic, Nilo-Saharan, and Khoisan, with each family predominantly used by certain populations across the African region. The Niger-Kordofanian family, spoken mainly by agriculturalists, covers a vast geographical distribution in Africa. Conversely, Afro-Asiatic is predominantly spoken by pastoralists and agro-pastoralists in northern and eastern Africa. Nilo-Saharan is the primary language spoken by Eastern and central African pastoralists, while Khoisan is primarily used by southern and eastern African hunter-gatherers [77,78].

The present study’s structure Analysis within African groups have unveiled further substructure, highlighting distinct genetic structures in north, east, west, and south Africa. These findings support the perception of substructure existing between cultures associated with hunter-gatherer and agriculturalist lifestyles [77,78].

The current study identified another noteworthy observation using STRUCTURE program, wherein a resemblant cluster pattern was observed between the four African population samples located outside of Africa and the populations from the west and south of the continent.

A logical explanation for this common genetic pattern could be attributed to the instances of the slave trade from Africa, which hold significant historical importance as one of the most prominent forced migration events. The transatlantic slave trade lasted for approximately five centuries, spanning from 1400 to 1900 and was categorized into four major waves. The final wave of the transatlantic slave trade, which primarily arose along the West African coasts, holds the distinction of being the largest in terms of scale and duration, with over 12 million Africans being coerced to undertake a perilous journey across the Atlantic Ocean [79].

North Africa’s demographic history has been characterized by distinctive features, such as population replacements, extensive gene flow, and diverse admixture from neighbouring regions, particularly the Middle East, which sets it apart from the rest of the continent. The Arab expansion, which began in the seventh century C.E., had a profound and lasting impact on North Africa that reached the westernmost part of the continent [80].

One of the debates revolves around the impact of Arabs on Berber populations, where two contrasting scientific theories exist. The first suggests that there is no genetic differentiation between Arab and Berber populations, as they share similar genetic characteristics [81,82]. The latter supports the notion that Arabs and Berbers have distinct gene pools [83]. Nevertheless, the present study’s findings do not unconditionally support either argument concerning the degree of genetic intermingling between Arab and indigenous Berber communities. Since, he Berbers of Morocco had a distinct genetic profile from Arabs, while Egyptian Berbers of Swia shared certain characteristics with Arabs in the Middle East. Further research is required to gain a deeper insight into the possibility of an Arab genetic influence on Berbers.

This study yielded significant insight into the genetic structure of Jewish communities. It was determined that, with the Middle Eastern Ashkenazi Jewish community being the sole exception, the Jewish populations in the study exhibited a genetic structure that is resemble of the Middle Eastern non-Jewish populations. This finding supports the notion of a shared regional ancestry among Jewish communities. Moreover, the results of the present study were consistent with those of other genome-wide analyses of Jewish populations [84].

The STRUCTURE analysis reveals that the Middle Eastern populations form a distinct cluster, corroborating prior investigations on a global scale [26]. The clustering can be attributed in part to the existence of microvariant alleles at the DYS458 markers [85] or maybe due to isolation and genetic drift in the region.

The Arabian Peninsula gene flow confirmed that all migration routes favoured divergence from ancestral populations without an ongoing migration model, which was the dominant model in this study. Furthermore, this study revealed that migration in the Arabian Peninsula occurred along coastal routes and the main migration route was from the United Arab Emirates to Kuwait and Iraq through Qatar. These findings agreed with previous research that looked at gene flow in the Arabian Peninsula as part of out-of-Africa migration [26]. Our gene flow findings are consistent with a previous genetic evaluation of the Qatari population, which discovered that the population has distinct genetic makeup coming from tribes in Southern Arabia [86]. Surprisingly, this corresponds to our own research findings, the migration trail from the United Arab Emirates to Qatar, which could possibly be the source of these genetic components.

## Supporting information

S1 Checklist Inclusivity in global research. (DOCX)

S1 Data Supporting data spread sheet contains: S1 Table: Y-STR haplotypes, and predicted Y-haplogroups for 379 Qatari [Arab] males. UP: unpredicted haplogroups. DYS389II.I represents the difference between the total repeat number at DYS389II and the repeat number at DYS389I. Fitness score (goodness of fit): a statistical measure that assesses how well a set of Y-STR (Y-chromosomal short tandem repeat) values align with the expected values for a specific haplogroup. Probability: a prediction or probability that a haplotype is in a particular haplogroup S2 Table: The quantification information for Qatari DNA samples. S3 Table: Allele frequency of Y-STR loci included in the PowerPlex Y23 kit in Qatari population. S4 Table: Forensic parameters of Y-STR loci included in the PowerPlex Y23 kit. The PowerPlex Y23 specific loci are highlighted in yellow. Gene diversity (GD), polymorphism information content (PIC), match probability (PM) and power of discrimination (PD). *The six loci not examined by the Yfiler kit are highlighted in yellow. S5 Table: Haplogroup Predictor results for the 379 Qatari male samples. *A1, *D, *H1, *O1 these haplogroups only appeared once. S6 Table: The matrix of pairwise genetic distance Rst of Y-STR between the Qatari population and the other Middle Eastern populations based on 17 Y-STR markers. S7 Table: Matrix plot showing population average pairwise differences based on 17 loci. Above diagonal: Average number of pairwise differences between populations (PiXY). Diagonal elements (yellow highlighted): Average number of pairwise differences within population (PiX). Below diagonal: Corrected average pairwise difference (PiXY-(PiX+PiY)/2). S8 Table: Estimation of migration rate in the Qatari population. (1) The migration routes between Qatar and Saudi Arabia; four models were tested. The most probable route was Qatar →Saudi, model 2. (2) The migration routes between Qatar and Yemen; four models were tested. The most probable route was the route Qatar →Yemen, model 2. (3) The migration routes between Qatar and Iraq; four models were tested. The most probable route was the route Qatar →Iraq, model 2. (4) The migration routes between Qatar and Kuwait; four models were tested and the most probable route was the route Qatar →Kuwait, model 2. 5. The migration routes between Qatar and the Emirates; four models were tested and the most probable route was the route Qatar → Emirates, model 2. (5) The migration routes between Qatar and the Emirates; four models were tested and the most probable route was the route Qatar → Emirates, model 2. (6) The most probable migration routes of each of the five countries were tested and the most probable migration route was Qatar →Yemen.
(XLSX)
